# Compliance with mandatory reporting of intimate partner violence among professionals in Norway

**DOI:** 10.1186/s12889-025-22637-z

**Published:** 2025-05-06

**Authors:** Christine Nordby, Kevin S. Douglas, Astrid Gravdal Vølstad, Thea Beate Brevik, Stål Kapstø Bjørkly, Solveig Karin Bø Vatnar

**Affiliations:** 1 Faculty of Health and Social Sciences, Molde University College, Molde, Norway; 2 Department of Psychology, Simon Fraser University, Burnaby, Canada; 3 Haukeland University Hospital, Centre for Research and Education in Forensic Psychiatry, Bergen, Norway; 4 Oslo University Hospital, Centre for Research and Education in Forensic Psychiatry, Oslo, Norway; 5 Faculty of Social Sciences and History, Volda University College, Volda, Norway

**Keywords:** Intimate partner violence, Mandatory reporting, Legal compliance

## Abstract

**Background:**

Mandatory reporting is a common legislative preventative measure for several types of crimes, among them family violence and specifically intimate partner violence (IPV). Among the individuals who are mandated to report under the law are professionals working with IPV victims and perpetrators in their practice. However, little is known about which characteristics are associated with compliance with the mandatory reporting of IPV (MR-IPV) law, on the one hand, and choosing not to report IPV, on the other.

**Methods:**

The current study sampled 357 professionals from 6 different agencies working with IPV victims and/or perpetrators. Six dichotomous outcome variables of compliance with MR-IPV and choosing not to report were analyzed by multiple logistic regression. The independent variables were professionals’ perceptions and knowledge of MR-IPV, context and workplace conditions, and experience with IPV cases and risk assessment.

**Results:**

Findings showed that risk of compliance with MR-IPV varied between complying *with* and *without* consent. Perceived applicability of MR-IPV for an IPV victim was the only variable that had significantly positively odds ratio for both compliance *with* and *without* consent. For choosing not to report, significant variables varied between whether the incident had taken place sometime throughout participants’ careers or during the last year, and whether it concerned a victim or a perpetrator. However, knowledge of MR-IPV, experience with IPV cases, expectations of MR-IPV, perceived workplace time management, and perception of compliance were significant for choosing not to report.

**Conclusions:**

Knowledge of the characteristics that are associated with professionals’ compliance with MR-IPV is essential to better understand the application of MR-IPV, to implement practice that is consistent with law, and ultimately to prevent IPV. Further research is needed to explore the context of compliance with MR-IPV.

**Supplementary Information:**

The online version contains supplementary material available at 10.1186/s12889-025-22637-z.

## Introduction

Intimate partner violence (IPV) is a serious public health issue that places people’s lives at risk [36], United Nations Women [UN Women] & United Nations Office on Drugs and Crime [UNODC], [43]). Although IPV can take various forms and degrees of severity, research has shown that previous victimization of IPV is one of the most consistent and strong risk factors for intimate partner homicide (IPH; e.g. [24, 38]. As such, recognizing IPV and preventing its escalation is considered pivotal to preventing IPH. One measure to prevent the potentially fatal harms of IPV is mandatory reporting of IPV (MR-IPV) and understanding the characteristics associated with professionals’ compliance with the law.

### Mandatory reporting of intimate partner violence

Mandatory reporting has been implemented internationally as a legal preventative approach for various crimes (e.g., *Australia:* [10], *Cyprus:* [30], *Netherlands:* [45], *United States of America* [23, 25]). The legal dictate for mandatory reporting varies between countries and states, but in its essence, it is a duty to report to authorities in order to prevent certain crimes before they occur or after they have occurred. For instance, several countries have mandatory reporting for civilians if they have evidence or suspicions of conspiracy to commit terrorism or attack against certain institutions (e.g., *France:* Code Pénal Article 434–1, [7]; *United Kingdom:* Terrorism Act Sect. 19, 2000 [41]). In other contexts, individuals might have a duty to report suspected abuse against children (e.g., *France:* Code Pénal Article 434–3, [7]; *India:* The Protection of Children from Sexual Offences Act Sect. 19, [42]; *Ireland*: Criminal Justice [Withholding of Information on Offences Against Children and Vulnerable Persons] Act Sect." Methods", [8]). Laws also vary according to whether only certain professionals are mandated to report, or civilians at large. To our knowledge, there is no comprehensive review of all MR-IPV laws internationally; however there are examples of other countries’ legislation available. For instance, in the USA, only a few states do not have any laws which mandate reporting due to suspicion or evidence of domestic violence (which includes but is not exclusive to IPV) (Are You Required to Report Domestic Violence?, Mandated Reporter, [3]). However nearly all states require professionals (and only professionals) to report to law enforcement when presented with a patient or client with physical wounds.

### MR-IPV in the Norwegian context

Although we are unable to verify all international law, to our knowledge, the Norwegian mandatory reporting law is unique on several points. Firstly, in Norway, where the current study was conducted, mandatory reporting applies to all individuals in the country, regardless of citizenship or residency, not just professionals. The law covers a duty to avert, not just report to police, several serious punishable offences under the Norwegian penal code, Sect. 196 [39], which refers to other sections within the code. The duty to avert, not just report, allows for a discretionary space where the individual who is averting might prevent future crime “through other means.” The type of “means,” however, is not further specified. Two of the offenses covered by Sect. 196 include IPV, called “abuse in close relations” (Sect. 282) and “aggravated abuse in close relationships” (Sect. 283). The types of acts that are defined as abuse, and hence make grounds for reporting, are “threats, force, deprivation of liberty, violence, and other degradations” [39]. Hence, even acts that are non-physical can be punishable and might need to be averted. These sections define current or former spouses or common-law partners among several types of “close relations.” Sect. 196 specifies that if an individual has information that leads them to believe that severe or repeated IPV is “certainly or most likely” occurring or will occur, they have a duty to attempt to avert the abuse or the consequences of it either through police reporting or by other means when/if this is still possible. Neglecting this duty is a punishable crime, and Sect. 196 clarifies that confidentiality does not relieve people from the duty to avert. In the context of MR-IPV, compliance means that individuals act according to their legal obligation to report/avert IPV. In brief, this means that anyone who has reasonable grounds to believe that IPV most likely will occur or be repeated is mandated to prevent or avert the IPV. In the current study, we define compliance as acting according to the law of MR-IPV.

### Compliance with MR-IPV

The World Health Organization (WHO) has discouraged MR-IPV [49, 50]. The concerns from WHO and researchers who are skeptical of MR-IPV center around the potential apprehension of IPV victims to seek medical care or other help if they are aware that professionals are obligated to report. However, it is important to highlight that in a systematic review of MR-IPV, empirical support for the recommendation was sparse and inconclusive [48]. Furthermore, the review uncovered a consistent pattern of emphasizing the results of a statistical minority who were against mandatory reporting over the views of the statistical majority who were in support of MR-IPV. The authors of the systematic review suggested that this was due to the researchers of the articles included in the review holding attitudes opposing MR-IPV.

A recent study of IPH found that a large majority of both victims (70%) and perpetrators (80%) of IPH had been in contact with one or more help-services prior to the homicide [46, 47]. Additionally, these authors found that half of the IPH cases had at least five independent documented incidents of previous IPV. This concurs with other international meta-analyses and systematic reviews [24, 38] that conclude that previous IPV is the best documented risk factor for IPH. Such findings indicate that there is a potential plethora of information within the help system that can be utilized to prevent IPV and IPH. The critical implications of this research on IPH are that professionals not only recognize risk factors and acknowledge their seriousness, but that they also forward this information and/or create interventions that can prevent further IPV and IPH [27, 34, 38]. However, some studies have found that professionals express several barriers to complying with the duty of reporting or otherwise intervening in cases of IPV [14, 22, 26, 37]. These barriers include lack of knowledge and/or evidence, lack of time to report, concerns for victim autonomy, and a perception of ineffective responses to reporting, all of which might prevent professionals from reporting, despite possibly being mandated to do so.

Research on IPV has shown that there are numerous risk factors for repeated violence and IPH (e.g., [20]), many of which professionals can be aware of and assess to potentially prevent subsequent violence. Many victims and perpetrators encounter professionals who could be mandated to report, and research has shown that not all information is always reported as required by law [47]. In fact, Vatnar and colleagues [47] found that identified risk of IPV only was communicated in 20% of total IPH cases they researched over an approximately 30-year period.

The research on compliance with MR-IPV is scarce and with only limited recent development [48]. Yet, there are some older studies exploring the phenomenon. One study examined health personnel in an emergency department setting who underwent a training program about guidelines on suspected IPV [2]. The researchers documented that of participants who reported having ever given care to a patient injured by IPV, only 23% had ever reported the IPV to the police. However, no potential correlates of reporting were investigated. Another study explored professionals’ compliance with their mandated duty to report IPV [31], yet the study only addressed *intent* to comply, hypothetically, and not actual compliance. Researchers asked 508 physicians if they would ever *not* report IPV to the police if the patient objected. There were several characteristics that significantly increased the odds ratio of choosing not to report: self-reported unawareness of the law; not attending any courses on IPV within the last three years; and type of medical practice (hospital based vs. health maintenance organization vs. private). Private practices and health maintenance organizations had increased odds ratio of 1.36 and 2.06 with respect to choosing not to report, respectively.

A recent qualitative study on Norwegian child welfare workers’ perceptions of MR-IPV found that participants’ understanding of the law was insufficient [9]. Further, although participants’ workplace primarily focused on the child’s welfare, child welfare workers in Norway are *also* mandated to report IPV. Despite this, participants incorrectly expressed, for instance, that this responsibility fell on others within the organization and was not within their legal responsibility. The child welfare workers also expressed that their responsibility was solely for the child. These findings suggest that compliance with the law might also be a consequence of insufficient understanding and knowledge of a professional’s duty and role. This might suggest insufficient or lack of teaching of the MR-IPV regulations in Norway for this professional group, or perhaps suggest specific work-place norms, although it is difficult to be conclusive without empirical grounds.

Despite the scarcity of compliance studies within MR-IPV, there are studies within related fields such as child abuse. Importantly, child abuse and IPV are comparable in some respects, but certainly not all. Some research has found that IPV can be perceived as a “private matter” [13], while children can be seen as more helpless and vulnerable [9, 15]. A study of reporting child abuse explored 200 Korean emergency nurses’ intentions to report [21]. The independent variables were based on the theory of planned behavior (TBP [1],) which posits that there are different components that predict intentions to behave or act (in this case, reporting equals the behavior). The components are taken from TBP,however, definitions were created for the context of the study by the authors. The expectations in terms of what would predict intentions to report were: perceived behavioral control, which was defined as confidence in reporting abilities and knowledge of reporting law; attitudes toward child abuse, defined as perception of responsibility to report; and knowledge of child abuse, defined as knowledge of child abuse symptoms and their severity. Attitudes toward child abuse had the largest effect and the model explained 22% of the variance in intentions to report.

A similar study, with the same theoretical framework as Lee and Kim [21], investigated 248 Saudi Arabian nurses’ intentions to report child abuse. They found a similar degree of explained variance (23%) after adding organizational support in guideline implementation as a component in their model [35]. However, this component is not included in the original model of theory of planned behavior by Ajzen [1]. By analyzing these components through multiple linear regression, knowledge about child abuse and reporting laws, subjective norms, and organizational support in guideline implementation were the only independent variables that were significant and showed an increase in intentions to report.

A qualitative study of 18 Israeli health care professionals, including pediatricians, nurses, social workers, and physio- and occupational therapists, explored mandatory reporting of child abuse [29]. Results showed that there were some facilitators that guided professionals to make correct decisions regarding reporting. The more suspicion grew, based on credibility of evidence or severity of injury to the child, the more likely participants were to express that they would report. They also noted that cooperation from the family would make it easier to report. The participants themselves expressed confidence in their knowledge of mandatory reporting of child abuse, but some expressed anxiety over making a wrong decision to the detriment of the child. All participants also expressed appreciation for having supervisors and colleagues to discuss cases with and having external experts they could contact for case guidance as well as emotional support. Not having access to risk assessment tools for abuse was mentioned as a barrier to reporting, as was working in small community settings with high likelihood of personal connections to cases.

### Aims and research questions

The aim of the current study was to explore if and how different contextual characteristics of professionals’ workplace conditions, knowledge and perceptions of MR-IPV, and experiences with IPV statistically predict having complied with MR-IPV or choosing not to report despite the potential risk of severe and repeated IPV. The law also includes a discretionary space where one might “avert through other means” rather than report directly to police, and over time one person might decide to report in some instances but not in others. Because of this, we also wanted to examine the instances where participants have not reported although there might have been reasons to suspect potential risk of IPV (i.e., the dependent variables: “choosing not to report”).

Our research questions were: Is self-reported compliance, as well as self-reported instances of choosing not to report, statistically predicted by the following characteristics?

- Perception and knowledge of mandatory reporting;
- Context and workplace conditions; and
- Professional experience with IPV cases and risk assessment.

## Methods

The current study is part of a larger project called [Identifying name], which examines awareness, attitudes, and experiences with MR-IPV among help seeking IPV victims or perpetrators and relevant groups of professionals in Norway. The project was approved by [Identifying ID] University Hospital’s Data Protection Official [Identifying ID]. Regional Committees for Medical Research Ethics (REK) deemed the study to be health service research, not health research, and hence not within their mandate [Identifying ID].

### Procedure

Researchers recruited participants to the current study from March 2022 until January 2023. Participants were recruited through in-person and digital meetings and seminars organized before the recruitment process commenced. These meetings and seminars were planned independently of the current study and organized by several national structures of the professional groups (i.e., National Police Directorate; The national board for IPV treatment centers; the umbrella organization for domestic violence shelters in Norway; National center for emergency primary health care). Researchers were given approximately 15 min to present the project and encouraged participants to participate. In person, participants were able to complete the questionnaire immediately after the presentation of the project. Most participants used between 45–50 min to complete the questionnaire, although with some variation. Digitally, participants were asked to contact the first author to receive a link to the questionnaire that they could complete in their own time. Invitations were sent to professionals in all regions of the country. All participants were duly informed that participation was voluntary, and all gave their informed consent to participate. The participants were given contact information to researchers in case they wished to withdraw, and their identity was concealed by cross-referencing their contact information on their consent form with a unique number in a separate file. Online, participants were given a randomly generated four-digit ID-code, which they could disclose to researchers if they wanted to withdraw (only one participant withdrew from the study). The total response rate was 86% (range across meetings: 36–100%; median 88%).

### Participants

A total of 357 participants were drawn from the following groups of professionals who work primarily or partly with IPV victims and/or perpetrators: police (*n* = 42, 12%), child welfare services (*n* = 36, 10%), emergency primary health care (*n* = 73, 20%), domestic violence shelters (*n* = 98, 28%), anger management treatment services (*n* = 66, 19%), and a treatment service called “Alternative to Violence” (*n* = 42, 12%). “Anger management” and “Alternative to Violence” both offer therapeutic treatment for perpetrators of violence. While “Alternative to Violence” offers treatment to clients directly from their clinics, “Anger management” is a therapy program offered by different professionals, among them public family welfare services. Because of these differences, these groups were separated in our analyses. Because the emergency department and sexual assault centers often work in tandem and generally consist of the same staff, we refer to these groups together as “emergency primary health care.” Notably, all of these professional groups have confidentiality regarding information they receive in their practice. However, the Norwegian MR-IPV law specifies that reporting IPV in the aim of averting future IPV is not considered a breach of confidentiality.

Most of the participants were female (79%), and no participants identified as anything other than male or female (missing: *n* = 15, 4%). The average participant was 45 years old (*SD* = 10; missing: *n* = 67, 19%) and had worked ten years (*SD* = 8) in their current position (missing: *n* = 35, 10%). Because there is a threshold in which mandatory reporting in the Norwegian law applies and we do not have the details of the specific cases from the participants to evaluate whether it would apply, participants who had no professional experiences with any cases of severe IPV or severe physical injury (*n* = 17) were excluded from all analyses. Although the law *might* apply in cases of IPV that do not involve severe IPV or severe physical injury, it also might not. Through this inclusion criterion, we sought to ensure that all participants included in the analyses had experienced at least one case of relevance to the MR-IPV law (see definition of abuse by Norwegian law in the introduction.)

### Measures

The questionnaire comprised unadjusted items from pre-existing questionnaires, adjusted items from pre-existing questionnaires, and newly developed items (see Additional file for translated survey.) For this study, items concerning attitudes toward guidelines, knowledge of mandatory reporting, and experience with MR-IPV were included. The adjusted items in the questionnaire originated from the questionnaire provided by the Norwegian Institute for Studies of the Medical Profession [12]. The original questionnaire has used repeated measurements on 2,200 doctors every other year since 1992 and varies between topics from year to year (e.g., [6, 32, 33]). The questionnaire covers Likert-scale statements on attitudes toward, awareness of, and experiences with different societal and organizational conditions, various ethical issues, and values. These items were adjusted to cover MR-IPV as a topic. The newly adjusted items addressed whether participants knew the law of mandatory reporting, experience with MR-IPV, knowledge of risk assessment tools for IPV, and perceived compliance with MR-IPV among other relevant professionals. A total of 59 items from the questionnaire were used for the current study, 6 of which were used to measure compliance and choosing not to report MR-IPV. The specific items used in this study are translated into English and presented in Additional File 1.

#### Compliance and choosing not to report

The dependent variables for this study comprised six variables within two categories (see Table S- 1, Additional File 1). One category used two items to measure compliance with MR-IPV, and the other four variables to measure choosing not to take action even in the face of risk of violence (henceforth called “choosing not to report”). For compliance, the items addressed how many cases participants had engaged in behaviors to avert IPV (with and without consent) from the IPV help-seeker in question; this item was dichotomized (0 vs. 1 or more). There was also a “not relevant” response option, but for the inferential statistical analyses, this option was treated as missing. For choosing not to report, participants were asked: “have you, as a professional, complied with a patient’s/client’s/user’s wish *not* to report or otherwise intervene to prevent IPV, even though you were unsure whether the patient/client/user understood the risk of violence related to their own situation, working with a person who (a) was victimized by IPV, or (b) has perpetrated IPV?” The participants were asked about cases with victims during the last 12 months *and* throughout career, and cases with perpetrators during the last 12 months *and* throughout career (hence, four items). All of these items have been used in a previous study [28].

It is noteworthy that the concept of “choosing not to report” does not necessarily mean that professionals would have violated the law even though they let someone go home to violence. The complexity and nuance within the legal text means that there might be instances where letting someone go home to potential violence for a time, while keeping note of the IPV perpetrator or victim as part of a larger plan to avert, could be within the discretionary part of the law *depending on the severity and risk for repeated IPV*. As such, it is more correct to describe these variables as choosing not to report through mandatory reporting rather than non-compliance per se (i.e., the opposite of the other dependent variables) as their actions might be within the law. Still, it is worth noting that individuals who choose to avert through other means bear greater legal responsibility to ensure that they have in fact averted the IPV, than if they had reported it directly to the police [16].

#### Perception and knowledge of mandatory reporting

##### Expectations about MR-IPV

Participants were asked about the expected consequences of MR-IPV for themselves and the help-seeker through nineteen items (see Table S- 2, Additional File 1). Some of these items included: mandatory reporting would have made it harder to work afterwards; mandatory reporting would have made me a more confident professional; there is a high likelihood that it would have had positive consequences for the patient/client/user; if I disclose confidential information, the patients/clients/users will lose trust in me, regardless of justification. These items were adjusted form the Norwegian Institute for Studies of the Medical Profession [12].

##### Perceived applicability of MR-IPV

Participants were asked how many IPV cases they have encountered where, in their opinion, MR-IPV applied with (a) a victim of IPV and (b) a perpetrator of IPV (see Table S- 2, Additional File 1).

##### Knowledge of mandatory reporting

Knowledge of mandatory reporting was measured by three items with a three-point scale. The questions addressed whether the professional knew the law of mandatory reporting generally and knew the law of mandatory reporting within their field of work, and if the professional had been informed about the criteria needed to make an evaluation of the applicability of mandatory reporting (see Table S- 2, Additional File 1). These items have also been used in Brevik et al.’ study [5] with a different sample and Nordby et al.’s study [28].

#### Context and workplace conditions

##### Perceptions of mandatory reporting compliance

Perceptions of mandatory reporting compliance was measured by four items. Each item asked the participants if it was their impression that MR-IPV was complied with in cases where there was a risk of harm. The first item asked about this without a specific context, whereas the others asked if it was their impression that (a) their leaders, (b) their colleagues, and (c) other agencies complied with MR-IPV (see Table S- 3, Additional File 1).

##### Perceptions of workplace time management

 The questionnaire also included six items about perception of time management in the workplace. Participants were asked how much time (i.e., one = no time; seven = a lot of time) they used on the following tasks in an average week: (a) work with patients/users/public/next of kin; (b) work-related meetings; (c) paper-work, phone calls, e-mail (including medical journals and similar); (d) professional development; (e) impractical organizational and practical working conditions, such as not achieving contact/appointment with other agencies, inadequate IT solutions, inefficient logistical solutions; and (f) organizational tasks they perceive as unnecessary, such as refilling paperwork, searching for relevant documents, unnecessary meetings (see Additional File 1).

##### Perception of workplace support

These questions concerned whether it is easy to bring up professional questions for discussion in the workplace; if it is okay to discuss professional disagreements; if disagreements are dealt with appropriately; if it is difficult to bring up unacceptable ethical behavior amongst colleagues; and if it is difficult to bring up unacceptable professional behavior amongst colleagues (see Table S- 3, Additional File 1). In inferential statistical analyses, the “not relevant” option was treated as missing.

#### Experience with IPV cases and risk assessment tools

##### Frequency of cases with types of IPV

We measured experience with IPV cases by asking how many times the participants had encountered victims and perpetrators, both for the past 12 months and career-wise, who were subjected to or had perpetrated IPV, severe IPV and IPV causing severe physical injury (see Table S- 4, Additional File 1). The number of encountered victims and perpetrators are referred to as “cases” in the results. Notably, according to the Norwegian legislation, severe IPV does not necessarily include physical injury as it can also include threats to harm or threats to kill. Consequently, we included a variable solely for severe physical injury. These items have also been used in Brevik et al.’s [5] study with a different sample and Nordby et al.’s [28] study.

##### Experience with risk assessment tools

The participants were asked about their experience with risk assessment tools through two items: (a) if they had ever completed some form of risk assessment, and (b) if they had ever completed a structured risk assessment for IPV (either tool, guideline, or manual; see Table S- 4, Additional File 1).

### Statistical analysis

The plan for statistical analysis relied on a multi-step variable screening approach (as according to [17]), based on significance (*p* ≤ 0.25), as suggested by Hosmer and Lemeshow [17]. First, we performed univariate logistic regression analyses for all the dependent and independent variables (see Tables 1, 2 and 3). In the second step, independent variables that were significant (*p* ≤ 0.25) in the univariate analyses were tested by category in multivariate analyses with all other significant variables within the same category (see Tables 4 and 5). These categories were not guided by a specific theory, but rather conceptually guided, meaning items that refer to the same concepts were categorized together. The items within the same category were tested in step 2 to adjust for conceptually similar items. There were nine groups of variables: a) Sociodemographic characteristics; b) Expectations of MR-IPV; c) Knowledge of MR-IPV; d) Perceived Applicability of MR-IPV; e) Perception of Mandatory Reporting Compliance; f) Perception of Workplace Support; g) Perception of Workplace Time Management; h) Frequency of Cases with Types of IPV; i) Experience with Risk Assessment. Finally, all variables that had remained significant (*p* ≤ 0.25) in the multivariate models were added into a final multivariate logistic regression model (see Tables 6 and 7).

**Table 1:** Logistic regression of sociodemographic characteristics of participants on compliance and choosing not to report

		Compliance		Choosing not to report			
				Victim		Perpetrator	
Item		w/o consent*	w/ consent†	Last 12 months‡	Throughout career**	Last 12 months††	Throughout career‡‡
Profession							
* OR *(Sig.)		.989 (.882)	.981 (.786)	1.136 (.**184**)	.978 (.769)	1.120 (.599)	.961 (.752)
CI		.863 - .1.135	.853 – 1.128	.941 – 1.373	.842 – 1.136	.734 – 1.711	.749 – 1.233
Years in position							
* OR *(Sig.)		1.015 (.302)	1.014 (.329)	1.012 (.514)	1.036 **(.021)**	.999 (.988)	1.053 **(.025)**
CI		.987 – 1.043	.986 – 1.043	.976 – 1.050	1.005 – 1.067	.924 1.081	1.006 – 1.101
Age							
* OR *(Sig.)		1.007 (.574)	.991 (.465)	1.026 **(.116)**	1.027 **(.041)**	1.077 **(.063)**	1.059 **(.010)**
CI		.984 – 1.030	.968 – 1.015	.993 – 1.059	1.001 – 1.054	.996 – 1.165	1.014 – 1.107
Gender							
* OR *(Sig.)		.938 (.822)	1.240 (.453)	1.294 (.533)	1.046 (.888)	1.785 (.588)	.478 **(.103)**
CI		.535 – 1.642	.706 – 2.179	.576 – 2.908	.562 – 1.946	.219 – 14.54	.197 – 1.159
County of work-place							
* OR *(Sig.)		1.000 **(.249)**	1.000 (.982)	1.000 (.822)	.999 (.442)	.999 (.574)	1.000 (.786)
CI		.999 – 1.001	.999 – 1.001	.999 – 1.001	.999 – 1.000	.997 – 1.002	.999 – 1.001

*N range = 287 - 351

*N range = 287 - 351

*‡N range = 282 - 347*

**N range = 277 - 343

*††N range = 278 - 340*

*‡‡N range = 272 - 331*

**Table 2:** Univariate logistic regression analyses of participants’ self-reported compliance with and without consent from the victim or perpetrator of IPV

	Compliance w/o consent*			Compliance w/ consent†		
Item	*OR*	Sig.	CI	*OR*	Sig.	CI
Experience with IPV						
IPV victim – career	1.193	**<.001**	1.130 – 1.259	1.162	**<.001**	1.102 – 1.227
IPV victim – 12 months	1.235	**<.001**	1.131 – 1.349	1.262	**<.001**	1.145 – 1.391
IPV perpetrator – career	1.101	**.001**	1.038 – 1.167	1.080	**.011**	1.018 – 1.147
IPV perpetrator – 12 months	1.095	**.191**	.956 – 1.254	1.037	.618	.899 – 1.196
Severe IPV victim – career	1.242	**<.001**	1.151 – 1.339	1.182	**<.001**	1.098 – 1.272
Severe IPV victim – 12 months	1.464	**<.001**	1.215 – 1.764	1.408	**<.001**	1.165 – 1.703
Severe IPV perpetrator – career	1.146	**.002**	1.054 – 1.247	1.068	**.109**	.985 – 1.158
Severe IPV perpetrator – 12 months	1.260	**.053**	.997 – 1.593	1.048	.685	.837 – 1.312
Severe physical injury victim – career	1.239	**<.001**	1.126 – 1.364	1.126	**.006**	1.034 – 1.225
Severe physical injury victim – 12 months	1.407	**.004**	1.112 – 1.780	1.149	**.169**	.943 – 1.401
Severe physical injury perpetrator - career	1.177	**.003**	1.057 – 1.309	1.081	**.121**	.979 – 1.194
Expectations about MR-IPV						
The incident would have been reviewed at the workplace	.683	**.072**	.451 – 1.034	.885	.558	.588 – 1.332
I would have been reproached by the patient/client/user/relatives afterwards	.797	.332	.505 – 1.259	.575	**.024**	.356 - .929
The patient/client/user would have less trust in me	.913	.671	.603 – 1.384	.599	**.021**	.388 - .925
There is a high probability that it would have had positive consequences for the patient/client/user	1.566	**.036**	1.029 – 2.382	2.29	**<.001**	1.484 – 3.542
	Compliance w/o consent			Compliance w/ consent		
Item	*OR*	Sig.	CI	*OR*	Sig.	CI
There is a high probability that it would have had negative consequences for the patient/client/user	.929	.726	.619 – 1.396	.710	**.109**	.468 – 1.079
All in all, the patient/client/user would have been better off	1.239	.274	.833 – 1.902	1.594	**.031**	1.044 – 2.434
It would have had few consequences for my patient/client/user	.812	**.229**	.579 – 1.139	1.011	.952	.717 – 1.424
I am very unsure what consequences it would have had for my patient/client/user	.801	**.146**	.594 – 1.079	.764	**.085**	.563 – 1.038
The MR-IPV case would have made it more difficult to work afterwards	.473	**.001**	.306 - .732	.491	**.001**	.318 - .757
The MR-IPV case would have had a negative impact on my private life	.648	**.066**	.407 – 1.029	.517	**.006**	.323 - .827
The MR-IPV case would have made me a more secure professional	1.057	.766	.732 – 1.528	1.315	**.152**	.904 – 1.911
The MR-IPV case had made me a more fearful professional	1.055	.802	.696 – 1.598	.607	**.020**	.398 - .924
The MR-IPV case would have few consequences for me personally	.855	.273	.647 – 1.131	.791	**.106**	.596 – 1.051
I would have received good and adequate support from the leaders at my workplace	.632	**.057**	.394 – 1.014	.901	.675	.567 – 1.429
I would have received good and adequate support from colleagues	.596	**.060**	.348 – 1.022	.849	.536	.506 – 1.426
I would have been confident that what I did was right	1.963	**.002**	1.288 – 2.992	2.267	**<.001**	1.481 – 3.469
Perceived applicability of MR-IPV						
Victim	2.122	**<.001**	1.689 – 2.667	2.431	**<.001**	1.849 – 3.194
vPerpetrator	1.809	**<.001**	1.412 – 2.319	1.663	**<.001**	1.299 – 2.127
Knowledge of MR-IPV						
Knowledge of MR-IPV	2.538	**<.001**	1.725 – 3.733	3.128	**<.001**	2.073 – 4.719
Knowledge of MR-IPV in field	2.884	**<.001**	1.979 – 4.202	2.961	**<.001**	2.025 – 4.329
Knowledge of criteria	3.355	**<.001**	2.277 – 4.945	3.354	**<.001**	2.269 – 4.959
Perceptions of MR-IPV compliance						
Compliance in general	1.527	**.080**	.950 – 2.453	2.027	**.004**	1.253 – 3.277
Compliance by leaders	1.417	**.187**	.844 – 2.376	1.839	**.022**	1.092 – 3.099
Compliance by colleagues	1.635	**.042**	1.018 – 2.625	1.581	**.054**	.992 – 2.519
Experience with risk assessment tools						
Some form of risk assessment	1.034	.666	.889 – 1.203	1.112	**.206**	.943 – 1.312
Structural risk assessment	1.511	**<.001**	1.228 – 1.859	1.374	**.002**	1.125 – 1.678
Perceptions of workplace time management						
Time with patients etc.	.850	**.019**	.743 - .973	.808	**.003**	.701 - .932
Impractical working conditions	.895	**.151**	.769 – 1.041	1.017	.832	.871 – 1.187

*Variables not significant: Experience with cases of severe physical injury with a perpetrator during the last 12 months; “The incident would have been reported to the supervisory health authorities”; “The patient/client/user would have created a less trusting relationship with the support system”; “The recipient of the message would have followed up on the message thoroughly”; Perceived compliance among other agencies; Perception of time spent on 1) meetings; 2) Paperwork, phone calls; emails etc.; 3) Unnecessary tasks; all items from “Perception of workplace support”*

*N range = 302 – 347

*†N range = 301 - 346*

**Table 3:** Univariate logistic regression analyses on participants’ self-reported choosing not to report regarding a victim and perpetrator within the last 12 months and throughout career

	Victim						Perpetrator					
	Last 12 months*			Throughout career†			Last 12 months‡			Throughout career**		
Item	*OR*	Sig.	CI	*OR*	Sig.	CI	*OR*	Sig.	CI	*OR*	Sig.	CI
Experience with IPV												
IPV victim – career	1.216	**<.001**	1.125 – 1.314	1.154	**<.001**	1.089 – 1.223	1.232	**.034**	1.016 – 1.494	1.149	**.006**	1.039 – 1.272
IPV victim – 12 months	1.208	**<.001**	1.117 – 1.307	1.119	**.002**	1.042 – 1.201	1.146	**.100**	.974 – 1.349	1.061	.329	.942 – 1.193
IPV perpetrator – career	1.043	**.234**	.973 – 1.119	1.057	**.061**	.997 – 1.120	1.193	**.016**	1.033 – 1.378	1.226	**<.001**	1.118 – 1.343
IPV perpetrator – 12 months	1.149	**.070**	.989 – 1.337	1.057	.459	.912 – 1.226	1.289	**.044**	1.007 – 1.651	1.336	**.004**	1.097 – 1.626
Severe IPV victim - career	1.176	**<.001**	1.094 – 1.263	1.157	**<.001**	1.085 – 1.234	1.119	**.151**	.959 – 1.304	1.107	**.047**	1.001 – 1.223
Severe IPV victim – 12 months	1.181	**.008**	1.045 – 1.335	1.185	**.008**	1.046 – 1.342	1.051	.739	.784 – 1.408	1.119	**.202**	.941 – 1.332
Severe IPV perpetrator – career	1.049	.302	.958 – 1.147	1.074	**.070**	.994 – 1.160	1.140	**.112**	.969 – 1.341	1.198	**.001**	1.081 – 1.327
Severe IPV perpetrator – 12 months	1.166	**.139**	.951 – 1.430	1.150	**.225**	.918 – 1.442	1.245	**.151**	.923 – 1.678	1.578	**.003**	1.172 – 2.126
Severe physical injury victim - career	1.274	**<.001**	1.173 – 1.383	1.173	**<.001**	1.087 – 1.266	1.210	**.011**	1.045 – 1.402	1.152	**.009**	1.037 – 1.280
Severe physical injury victim – 12 months	1.569	**<.001**	1.257 – 1.960	1.396	**.002**	1.133 – 1.723	1.193	**.196**	.913 – 1.559	1.119	.360	.879 – 1.423
Severe physical injury perpetrator - career	1.071	**.180**	.969 – 1.183	1.094	**.049**	1.000 – 1.197	1.179	**.053**	.998 – 1.395	1.188	**.004**	1.058 – 1.335
Severe physical injury perpetrator – 12 months	1.204	**.135**	.944 – 1.536	1.258	**.128**	.936 – 1.691	1.284	**.086**	.965 – 1.708	1.476	**.014**	1.081 – 2.016
Expectations about MR-IPV												
I would have been reproached by the patient/client/user/relatives afterwards	.657	**.158**	.367 – 1.177	.683	**.122**	.421 – 1.108	1.245	.748	.328 – 4.719	.751	.473	.343 – 1.644
There is a high probability that it would have had positive consequences for the patient/client/user	1.455	**.196**	.824 – 2.569	1.051	.829	.667 – 1.657	1.458	.556	.415 – 5.126	1.128	.753	.531 – 2.397
I am very unsure what consequences it would have had for my patient/client/user	1.033	.872	.695 – 1.536	.958	.793	.694 – 1.323	2.558	**.087**	.872 – 7.506	1.225	.469	.707 – 2.121
The MR-IPV case would have made it more difficult to work afterwards	.793	.431	.445 – 1.412	.724	**.181**	.451 – 1.162	1.195	.762	.376 – 3.799	1.024	.950	.489 – 2.145
The MR-IPV case would have had a negative impact on my private life	.668	**.237**	.343 – 1.304	.674	**.148**	.395 – 1.149	.656	.589	.143 – 3.019	.716	.471	.289 – 1.776
The MR-IPV case would have made me a more secure professional	1.760	**.039**	1.029 – 3.010	1.159	.473	.774 – 1.735	2.175	**.237**	.601 – 7.877	1.377	.370	.684 – 2.769
The MR-IPV case would have made me a more fearful professional	.582	**.097**	.307 – 1.102	.629	.**065**	.386 – 1.028	1.333	.610	.442 – 4.019	.737	.463	.326 – 1.666
The MR-IPV case would have few consequences for me personally	.557	**.005**	.369 - .840	.759	**.081**	.558 – 1.035	.689	.403	.288 – 1.649	1.015	.954	.617 – 1.668
I would have received good and adequate support from the leaders at my workplace	.981	.949	.548 – 1.758	1.053	.833	.649 – 1.707	††	††	††	1.925	**.202**	.704 – 5.268
I would have received good and adequate support from colleagues	.896	.742	.465 – 1.727	1.508	**.193**	.812 – 2.800	1.633	.610	.248 – 10.752	4.761	**.115**	.683 – 33.183
Perceived application of MR-IPV												
Victim	1.170	**.002**	1.059 – 1.293	1.175	**.001**	1.069 – 1.293	1.017	.899	.780 – 1.327	1.142	**.046**	1.002 – 1.301
Perpetrator	1.142	**.055**	.997 – 1.308	1.134	**.052**	.999 – 1.288	1.147	.275	.897 – 1.467	1.134	**<.001**	1.134 – 1.539
Knowledge of MR-IPV												
Knowledge of MR-IPV	1.999	**.006**	1.218 – 3.282	1.885	**.002**	1.255 – 2.831	.745	.576	.265 – 2.095	1.641	**.142**	.848 – 3.178
Knowledge of MR in field	1.433	**.144**	.884 – 2.323	1.427	**.075**	.965 – 2.110	.926	.879	.345 – 2.488	1.396	.322	.722 – 2.699
Knowledge of criteria	1.829	**.014**	1.130 – 2.959	1.409	**.075**	.966 – 2.054	.845	.731	.324 – 2.204	1.176	.611	.630 – 2.192
Perceptions of MR-IPV compliance												
Compliance in general	.821	.514	.455 – 1.483	.716	**.182**	.439 – 1.169	.537	.265	.180 – 1.602	.917	.835	.406 – 2.072
Compliance by leaders	.643	**.148**	.353 – 1.170	.823	.476	.481 – 1.407	.266	**.006**	.105 - .679	.703	.383	.319 – 1.550
Compliance by colleagues	.633	**.098**	.368 – 1.088	.786	.328	.485 – 1.273	.151	**<.001**	.0628 - .363	.564	**.096**	.287 – 1.107
Compliance by agencies	.792	.369	.476 – 1.317	.619	**.028**	.404 - .949	.451	**.158**	.149 – 1.362	.819	.570	.412 – 1.629
Experience with risk assessment tools												
Experience with risk assessment tools	1.178	**.069**	.987 – 1.407	1.120	.161	.956 – 1.313	1.206	**.243**	.881 – 1.651	1.100	.425	.870 – 1.391
Perceptions of workplace time management												
Work with patients etc.	1.059	.535	.883 – 1.269	1.049	.515	.909 – 1.211	1.281	.275	.821 – 1.998	.849	**.128**	.687 – 1.048
Meetings	1.106	.291	.917 – 1.333	1.045	.577	.896 – 1.219	1.494	**.038**	1.022 – 2.185	1.194	**.144**	.941 – 1.515
Paperwork, phone calls, emails, etc.	1.086	.400	.896 – 1.317	1.009	.914	.863 – 1.178	1.277	**.227**	.859 – 1.899	.939	.622	.733 – 1.204
Professional development	1.100	.421	.872 – 1.388	1.166	**.120**	.961 – 1.415	.972	.912	.592 – 1.598	1.231	**.153**	.926 – 1.636
Impractical working conditions	1.267	**.013**	1.050 – 1.528	1.249	**.007**	1.063 – 1.467	1.212	.309	.837 – 1.755	1.039	.768	.804 – 1.343
Unnecessary tasks and meetings	1.161	**.140**	.952 – 1.416	1.178	**.057**	.995 – 1.394	1.079	.716	.716 – 1.625	1.015	.912	.775 – 1.331

*Variables not significant: “The incident would have been reported to the supervisory health authorities”; “The incident would have been reviewed at the workplace”; “The patient/client/user would have less trust in me”; “The patient/client/user would have created a less trusting relationship with the support system”; “There is a high probability that it would have had negative consequences for the patient/client/user”; “All in all, the patient/client/user would have been better off”; “It would have had few consequences for my patient/client/user”; “The recipient of the message would have followed up on the message thoroughly”; “I would have been confident that what I did was right”; Experience with some form of risk assessment; all items from “Perception of workplace support”*

†† *This result was omitted in analysis*

*N range =  292 - 343

*†N range = 292 - 342*

*‡N range = 252 - 338*

**N range = 283 - 330

**Table 4:** Category multiple logistic regression analyses on self-reported compliance with and without consent from the victim or perpetrator of IPV

Item	Compliance w/o consent*			Compliance w/ consent†		
	*OR*	Sig.	CI	*OR*	Sig.	CI
Experience with IPV						
IPV victim – career	1.132	**.040**	1.006 – 1.273	1.138	**.039**	1.007 – 1.286
IPV victim – 12 months	1.093	.344	.909 – 1.314	1.208	**.059**	.993 – 1.471
IPV perpetrator – 12 months	.709	**.045**	.507 - .993			
Severe physical injury victim – 12 months	.924	.658	.651 – 1.311	.719	**.051**	.516 – 1.001
Expectations about MR-IPV						
The incident would have been reviewed at the workplace	.727	**.198**	.448 – 1.181			
There is a high probability that it would have had positive consequences for the patient/client/user	1.243	.378	.767 – 2.015	1.755	**.048**	1.005 – 3.064
It would have had few consequences for my patient/client/user	.801	**.232**	.557 – 1.152			
The MR-IPV case would have made it more difficult to work afterwards	.443	**.005**	.252 - .779	.695	**.194**	.401 – 1.204
The MR-IPV case would have few consequences for me personally				.828	**.222**	.611 – 1.121
I would have received good and adequate support from the leaders at my workplace	.592	**.125**	.304 – 1.156			
I would have received good and adequate support from colleagues	.612	**.198**	.289 – 1.294			
I would have been confident that what I did was right	2.131	**.005**	1.257 – 3.612	1.676	**.038**	1.029 – 2.729
Perceived application of MR-IPV						
Victim	1.907	**<.001**	1.478 – 2.459	2.489	**<.001**	1.782 – 3.476
Perpetrator	1.236	**.168**	.915 – 1.671	.972	.866	.704 – 1.344
Knowledge of MR						
Knowledge of MR	1.086	.782	.603 – 1.957	1.519	**.174**	.831 – 2.777
Knowledge of MR in their field	1.687	**.072**	.954 – 2.983	1.496	**.169**	.843 – 1.654
Knowledge of criteria	2.382	**<.001**	1.478 – 3.837	2.144	**.002**	1.329 – 3.456
Perceptions of MR-IPV compliance						
Compliance in general	1.328	.387	.698 – 2.527	1.769	**.084**	.927 – 3.375
Compliance by colleagues	1.678	**.151**	.827 – 3.401	1.029	.937	.508 – 2.086
Experience with risk assessment tools						
Structural risk assessment				1.364	**.003**	1.111 – 1.674
Perceptions of workplace time management						
Time with patients etc.	.865	**.042**	.753 - .995			

*N range = 302 – 335

*†N range = 324 – 334*

**Table 5:** Category multiple logistic regression analyses of participants self-reported choosing not to report regarding a victim and perpetrator within the last 12 months and throughout career

	Victim						Perpetrator					
	Last 12 months*			Throughout career†			Last 12 months‡			Throughout career**		
Item	*OR*	Sig.	CI	*OR*	Sig.	CI	*OR*	Sig.	CI	*OR*	Sig.	CI
Sociodemographic characteristics												
Gender										.448	**.096**	.174
Profession	1.179	**.137**	.949									
Year in position				1.024	**.210**	.9867 – 1.063				1.014	.605	.961 – 1.070
Age	1.023	**.181**	.989 – 1.057	1.0217	**.197**	.9889 – 1.055				1.061	**.027**	1.007 – 1.117
Experience with IPV												
IPV victim – career	1.158	**.029**	1.015 – 1.319	1.175	**.003**	1.056 – 1.307	1.049	.754	.778 – 1.415	.965	.728	.788 – 1.181
IPV victim – 12 month	1.124	**.156**	.956 – 1.321	.947	.437	.826 – 1.086	1.037	.808	.772 – 1.395			
IPV perpetrator – career	.913	**.234**	.785 – 1.061	.947	.355	.845 – 1.063	1.261	**.161**	.912 – 1.742	1.459	**.001**	1.163 – 1.831
IPV perpetrator – 12 months	1.369	**.103**	.939 – 1.995				.964	.921	.469 – 1.983	.559	**.040**	.321 – .973
Severe IPV victim – 12 months	.752	**.107**	.531 – 1.064	1.073	.392	.914 – 1.259				.931	.591	.716 – 1.209
Severe physical injury victim - career	1.215	**.043**	1.006 – 1.466	.979	.805	.834 – 1.151	1.362	**.049**	1.001 – 1.851	1.209	**.096**	.967 – 1.511
Severe physical injury victim – 12 months	1.549	**.031**	1.040 – 2.306	1.262	**.155**	.916 – 1.739	.813	.558	.406 – 1.626			
Severe physical injury perpetrator – career										.788	**.216**	.539 – 1.149
Severe physical injury perpetrator – 12 months							2.159	**.233**	.609 – 7.659	2.333	**.081**	.902 – 6.037
Expectations about MR-IPV												
I am very unsure what consequences it would have had for my patient/client/user							3.023	**.046**	1.028 – 8.974			
The MR-IPV case would have made me a more secure professional	1.808	**.044**	1.017 – 3.212				2.947	**.110**	.782 – 11.109			
The MR-IPV case would have few consequences for me personally	.593	**.012**	.396 - 891	.812	**.188**	.595 – 1.107						
I would have received good and adequate support from colleagues										4.280	**.173**	.528 – 34.707
Perceived application of MR-IPV												
Victim	1.168	**.011**	1.036 – 1.316	1.179	**.005**	1.052 – 1.323				.947	.642	.752 – 1.192
Perpetrator	1.017	.841	.863 – 1.198	1.007	.929	.864 – 1.173				1.377	**.008**	1.086 – 1.746
Knowledge of MR-IPV												
Knowledge of MR-IPV	2.459	**.033**	1.073 – 5.637	2.145	**.018**	1.140 – 4.035						
Knowledge of MR in field	.524	**.130**	.227 – 1.209	.783	.440	.419 – 1.458						
Knowledge of criteria	1.555	**.184**	.811 – 2.979	1.027	.916	.614 – 1.690						
Perceptions of MR compliance												
Compliance in general				.859	.583	.500 – 1.476						
Compliance by leaders	.865	.754	.349 – 2.144				1.136	.848	.308 – 4.187			
Compliance by colleagues	.698	.391	.306 – 1.588				.086	**<.001**	.022 - .339			
Compliance by other agencies				.653	**.074**	.409 – 1.042	2.412	.315	.434 – 13.419			
Perceptions of workplace time management												
Time spent with patients etc										.819	**.069**	.661 – 1.016
Meetings							1.498	**.107**	.917 – 2.447	1.158	.278	.888
Impractical working conditions	1.371	**.045**	1.006 – 1.867	1.269	**.072**	.979 – 1.648						
Unnecessary tasks and meetings	.897	.519	.646 – 1.247	.951	.720	1.254						

*N range =  282 - 333

*†N range = 254 - 328*

*‡N range = 296 - 320*

**N range = 249 - 320

**Table 6:** Final multiple logistic regression model of self-reported mandatory reporting of intimate partner violence compliance with and without consent from the victim or perpetrator of intimate partner violence

Item	Compliance w/o consent*			Compliance w/ consent†		
	*OR*	Sig	*CI*	*OR*	Sig	*CI*
County of workplace	1.000	.565	.999 – 1.002			
Experience with IPV						
IPV victim – career	1.056	.256	962 – 1.159	1.025	.632	.926 – 1.135
IPV victim – 12 months				1.135	.229	.924 – 1.394
IPV perpetrator – 12 months	.938	.584	.741 – 1.184			
Severe physical injury victim – 12 months				.784	.094	.589 – 1.042
Expectations about MR-IPV						
The incident would have been reviewed at the workplace	.772	.434	.404 – 1.476			
There is a high probability that it would have had positive consequences for the patient/client/user				1.459	.219	.799 – 2.668
It would have had few consequences for my patient/client/user	.860	.517	.546 – 1.356			
The MR-IPV case would have made it more difficult to work afterwards	.615	.167	.308 – 1.226	.973	.931	.527 – 1.798
The MR-IPV case would have few consequences for me personally				.779	.227	.520 – 1.168
I would have received good and adequate support from the leaders at my workplace	.493	.175	.178 – 1.368			
I would have received good and adequate support from colleagues	.494	.187	.173 – 1.408			
I would have been confident that what I did was right	1.396	.302	.741 – 2.630	1.228	.486	.689 – 2.185
Perceived applicability of MR-IPV						
Victim	1.407	**.043**	1.011 -1.959	1.989	**<.001**	1.408 – 2.811
Perpetrator	1.617	**.015**	1.097 – 2.385			
Knowledge of MR-IPV						
Knowledge of MR				1.227	.620	.546 – 2.755
Knowledge of MR in their field	1.464	.221	.795 – 2.696	1.719	.144	.831 – 3.556
Knowledge of criteria	1.694	.107	.892 – 3.219	1.518	.197	.805 – 2.861
Perceptions of MR-IPV compliance						
Compliance in general				3.379	**<.001**	1.704 – 6.703
Compliance by colleagues	1.924	.070	.947 – 3.909			
Experience with risk assessment tools						
Structural risk assessment	1.205	.114	.956 – 1.517	1.118	.312	.901 – 1.388
Perceptions of workplace time management						
Time with patients etc.	.904	.114	.956 – 1.517	.807	**.024**	.669 - 973

*N  = 261

*†N = 270*

**Table 7:** Final multiple logistic regression model of choosing not to report intimate partner violence on behalf of the victim or perpetrator of intimate partner violence during the last 12 months or throughout their career

	Victim						Perpetrator					
	Last 12 months*			Throughout career†			Last 12 months‡			Throughout career**		
Item	*OR*	Sig	*CI*	*OR*	Sig	*CI*	*OR*	Sig	*CI*	*OR*	Sig	*CI*
Experience with IPV												
IPV victim – career	1.334	.133	.962 – 1.336	1.098	**.048**	1.001 – 1.204						
IPV perpetrator – career	.857	.078	.722 – 1.017				1.352	.069	.977 – 1.870	1.286	**.013**	1.055 – 1.567
Severe physical injury victim - career	1.153	.225	.916 – 1.451				1.667	**.020**	1.083 – 2.565	1.178	.099	.969 – 1.431
Expectations about MR-IPV												
The MR-IPV case would have made me a more secure professional	1.822	.112	.869 – 3.816									
The MR-IPV case would have few consequences for me personally	.521	**.024**	.296 – .918	.777	.231	.514 – 1.174	1.230	.774	.299 – 5.047			
Perceived applicability of MR-IPV												
Victim	.937	.528		1.025	.729	.889 – 1.182						
Perpetrator										1.057	.641	.838 – 1.332
Knowledge of MR-IPV												
Knowledge of MR-IPV	.405	1.45	.119 – 1.367	1.892	**.049**	1.002 – 3.573				1.578	.341	.617 – 4.033
Knowledge of criteria	2.651	**.049**	1.004 – 7.001									
Perceptions of MR compliance												
Compliance by colleagues							.028	**.003**	.003 - .301	.544	.187	.220 – 1.345
Perceptions of workplace time management												
Time spend with patients etc.										.741	**.040**	.556 - .987
Work-related meetings							1.358	.338	.726 – 2.538			
Impractical working conditions												

*N = 242

†*N* = 223

‡*N *= 244

**N = 236

Given the cross-sectional nature of analyses, any reference to “prediction” in our results refers to statistical prediction only and does not imply time-ordered associations. Multicollinearity was checked for all independent variables against all dependent variables. No independent variable reached a Variance Inflation Factor (VIF) above 10 and mean VIF ranged between 3.18 and 3.21. A Hosmer–Lemeshow test was used to test model fit for all models. Responses where participants had responded in between options or indicated two options to one question were imputed using the Bernoulli method of imputation. Analyses were performed using the statistical program Stata version 18.

## Results

### Univariate and category logistic regression results for compliance and choosing not to report

Several items were significant in both the univariate and category multivariate analyses. Because of the large number of variables and analyses performed in this study details regarding results from the initial analyses are only presented in Tables 1, 2, 3, 4 and 5. These tables present an overall view of significant variables highlighted and variables selected for the category multivariate models and final multivariate models. In addition, these tables provide insight into the differences between findings for compliance and for choosing not to report. There were variables that were significant only for one of the compliance variables, and only variables concerning time of incident (i.e., last 12 months or throughout career), however, the following results include significant findings for both of the compliance variables and for both the victim *and* both perpetrator variables, as these are perceived to be the most important findings in the initial stages and for brevity.

Some sociodemographic variables were significant in the univariate analyses. Only county of workplace was significant for compliance without consent (hence included in the final model, see Table 6); none were significant for compliance with consent. The sociodemographic variables significant for choosing not to report were higher age (for all four variables), greater number of years in position (for victim and perpetrator throughout career), profession (for victim during the last 12 months), and gender (females less likely to choose not to report) (for perpetrator throughout career). None were significant in the final models; hence they will not be described in detail (see Table 1 for results.)

In the univariate analyses 50 of 58 variables were significant for at least one of the dependent variables. The variables that were significant for all dependent variables were related to the professionals’ experience with IPV cases, such as all cases throughout career, specifically: cases with 1) victims and 2) perpetrators of IPV; 3) victims of severe IPV; and 4) victims and 5) perpetrators of severe physical injury (see Tables 2 and 3 for odds ratio and *p*-value.) The only variable that was significant for both compliance variables, but not for the variables for choosing not to report was for the item “*I would have been confident that what I did was right*” which had a positive odds ratio for both items (see Table 2). Only one variable was significant for all variables regarding choosing not to report that was not significant for any of the compliance variables. This variable was experience with cases of a perpetrator of severe physical injury during the last 12 months (see Table 3). Beyond this, no variables were only significant for choosing not to report.

However, there were some differences between choosing not to report regarding a victim or a perpetrator. Choosing not to report regarding a victim in the univariate analyses was significantly positively predicted by knowing the law in their field and knowing the criteria of MR-IPV, and by impractical working conditions and unnecessary tasks and meetings. Choosing not to report regarding a victim was negatively predicted by the following items regarding expectations of MR: “*I would have been reproached by the patient/client/user/relatives afterwards;” “The MR-IPV case would have had a negative impact on my private life;” “ The MR-IPV case would have made me a more fearful professional;”and “The MR-IPV case would have few consequences for me personally.”* Notably, only one variable was significant for choosing not to report regarding a perpetrator that was not significant for the victim variables. This item was the perception of time spent on meeting activities, which had a positive odds ratio, meaning participants who perceived they spent more time on this had higher risk of choosing not to report regarding a perpetrator.

In the category analyses, 33 variables were significant, but no variable was significant for all dependent variables (see Table 4). However, six variables were significant for both compliance variables. Compliance was still positively predicted by experience with cases of IPV victims throughout career, the perception that they would have been confident in what they did was right; perceived application of MR regarding a victim (but only positively predicted by the perpetrator item for compliance without consent), and finally both knowing the law in their field and the criteria of MR-IPV. Additionally, both were negatively predicted by the perception that the case would make it more difficult to work afterwards.

Choosing not to report regarding both the victim variables was positively predicted by professionals’ experience with cases of both IPV victim throughout career and victims of severe physical injury during the last 12 months, as well as perceived application of MR-IPV regarding a victim, knowing the MR law in general, and perceived inappropriate workplace organization (see Table 5). Choosing not to report regarding a victim were only negatively predicted by the perception that the MR-IPV case would have few consequences for them personally. Choosing not to report regarding both the perpetrator variables was only predicted by experience with cases of perpetrators of severe physical injury during the last 12 months, with positive odds ratio.

### Compliance with MR-IPV in final multivariate models

Perceived application of MR-IPV regarding a victim was a common significant variable for both compliance with and without consent, both with positive odds ratios. Moreover, the perceived application of MR-IPV regarding a perpetrator was significant but only for compliance *without* consent, also with a positive odds ratio. Time spent with patients, relatives, the public, etc., was significant with a negative odds ratio, as well as perceived compliance in general with a positive odds ratio for compliance *with* consent. Other variables were tested in the final model but were not significant (see Table 6).

### Choosing not to report in final multivariate models

For choosing not to report there were differences in which variables remained significant in the final multivariate model depending on whether not reporting was with respect to a victim or a perpetrator, and depending on timing of incident (i.e., past 12 months vs lifetime) (see Table 7). For choosing not to report regarding a victim, knowledge of MR had significant positive odds ratios, although there were different significant knowledge items for choosing not to report during the last 12 months (knowledge of criteria) and throughout career (knowledge of MR in general)**.** In addition, the perception that the case would have few consequences for the participant had a significant negative odds ratio for choosing not to report regarding a victim during the last 12 months. Experience with cases of IPV victims throughout career had a significant positive odds ratio for choosing not to report regarding a victim throughout career.

Regarding the professionals choosing not to report an IPV perpetrator, the professionals’ experience with IPV cases was significant for both the last 12 months and throughout career (see Table 7). Specifically, experience with cases of victims of severe physical injury throughout career had positive odds ratio for choosing not to report during the last 12 months, while experience with cases of perpetrators of IPV throughout career had significant positive odds ratio for choosing not to report a perpetrator throughout career. Perceived compliance by colleagues had significant negative odds ratio on choosing not to report regarding a perpetrator during the last 12 months, and time spent with patients, relatives, and/or public, etc., had significant negative odds ratio on choosing not to report throughout career.

## Discussion

The results showed several characteristics of the professional’s work experience and context as predictors of their compliance with MR-IPV and choosing not to report even in the face of risk of violence.

### Compliance with MR-IPV

In the final multivariate model, perceived applicability of MR-IPV with respect to victims, in both dependent variables, and for perpetrator in compliance *without* consent, remained significant predictors of compliance with MR-IPV. This finding is intuitively understandable, as the more cases one perceives to have been relevant to report, the more likely one is to have actually reported under MR-IPV. Interestingly, perceived applicability of MR-IPV regarding a *perpetrator* was only significant in the final model for compliance without consent. It might be that perpetrators relative to victims are generally less likely to give consent to a professional. Kristiansen et al. [19] found in their interviews with IPV perpetrators who had experienced MR-IPV that the perpetrators found the professionals’ interventions unnecessary and experienced it as a breach of trust. Although this study did not look specifically at perpetrators’ likelihood of giving consent, their responses do suggest perpetrators are somewhat opposed to MR-IPV by professionals. It is worth noting that the items for compliance did not distinguish between compliance regarding a victim and regarding a perpetrator, so it cannot be concluded for sure that the case(s) of compliance that the participants refer to were regarding victims or perpetrators. However, the current study included both professionals who mainly worked with perpetrators and those who worked mainly with victims, suggesting that there should have been a mix of cases with both.

The results might also imply that when professionals considered MR-IPV to be applicable, they were mainly influenced by the potential risk to the victim (even professionals who mostly worked with perpetrators). The danger for repeated violence or escalation of IPV to IPH might also be clearer in light of an IPV victim’s information compared to a perpetrator’s, which could be why this item was still significant for both dependent variables. A couple of studies have found that within couples (either current or former partners) the perpetrating partner generally reports fewer occurrences and types of IPV than their female partners [40, 44]. In addition, professionals applying MR-IPV can have very different consequences for IPV perpetrators and victims. For the IPV victims, MR-IPV is a measure only intended to safeguard and help. For IPV perpetrators, MR-IPV might also lead to police involvement and safety measures that are perceived as negative for the IPV perpetrator. Professionals working with IPV victims and perpetrators might be affected by this difference in the consequences for their clients and therefore act differently.

Additionally, perceived general compliance was a relatively strong predictor of compliance with consent. There might be a social psychological explanation as to why perception of others’ compliance is relevant for one’s own compliance. Social psychological theories of group influence on individual decision-making, for instance normative influence [18], suggests that individuals will conform to the group norm in order to avoid social disapproval. In this instance, although other characteristics were found to be relevant for complying, it would seem the perception of general compliance with MR-IPV was the strongest influence on one’s own compliance. However, it is uncertain why this was only the case for complying in cases where consent was present. It is possible that complying without consent is more difficult for professionals to do, as they might fear a breach of trust, so complying without consent is more reliant on the perception of whether MR-IPV truly applies to the case relative to other characteristics.

Also, professionals who spent more time on work with patients/relatives/public overall had significantly decreased odds ratio of complying with MR-IPV with consent (see Table 6). The explanation for this finding is not immediately clear. It could be attributable to the fact that more time spent with patients or users/public/next of kin means that the professionals have less time to evaluate the risk of IPV and consequently MR-IPV because it means they have a lot of individuals they have to see to during a day and consequently less time for risk assessment, but future research would be needed to address this possibility.

### Choosing not to report

For choosing not to report, interestingly, knowledge of MR was positively predictive for victims, but not for perpetrators. It is worth noting that the items refer more to confidence in knowledge than actual knowledge, as the measures did not verify that their knowledge was correct. Still, this is an interesting finding. On a general note, it is possible that confidence in their knowledge about MR-IPV also allows professionals to more confidently evaluate and choose to not report, even in the face of risk for severe or repeated IPV, because they have more accurate knowledge of when it is more relevant or important to report to the police. Balancing between reporting to the police and averting through other means can be tricky, and it could be the case that professionals who were less confident found it safer just to report to the police or even had another colleague reporting it. More importantly, considering this result was only significant regarding victims, it might imply that professionals feel they need to be more confident in or know the law better to feel confident in choosing not to report regarding an IPV victim. Specifically, there is greater risk of harm for a victim than for a perpetrator. MR-IPV certainly has very different outcomes for a victim than for a perpetrator, and it might be that regardless of confidence in knowledge, professionals who work with perpetrators allow their clients to go home because there is a risk of breaching trust in the relationship and the perpetrator might be prosecuted in the most extreme case.

In addition, increased number of cases of IPV victims throughout the participant’s career was significant for choosing not to report regarding a victim throughout career. As with compliance with MR-IPV, increased frequency of cases could allow more opportunity not to report because participants might have experienced more cases where they might have chosen not to report than participants with less experience IPV cases. It is, however, difficult to explain why this was not significant for choosing not to report during last 12 months.

Lastly, the perception that the case would have few consequences was significant for choosing not to report regarding a victim during the last 12 months. This item had decreased odds ratio, meaning that if a professional responded that reporting would have few consequences for them, there was a lower risk of choosing not to report. It might be that professionals who believe a case would have few consequences are more comfortable with acting on the suspicion that the victim or perpetrator might not understand the risk of their situation. Perhaps the consequences they fear are from acting on a suspicion of danger, but participants who are more distanced from these concerns are less likely to report. There is no definitive conclusion that this is the case, but other studies have also found that feeling confident in one’s abilities and knowledge of mandatory reporting predict compliance with mandatory reporting [21, 35].

For the perpetrator variables, two items from experience with cases of IPV were significant. The more professional experience with cases of IPV perpetrators, the higher risk of having chosen not to report a perpetrator throughout their career. Again, the argument can be made that this implies more opportunities to evaluate the use of MR-IPV and subsequently choosing not to report, even in face of risk of future IPV. In addition, professionals’ number of cases with severe physical injury to an IPV victim was a significant positive predictor for choosing not to report (during the last 12 months). It might be that, coincidentally, there was a significant number of participants who chose not to report during the last 12 months before participating in this study who also had cases with victims of severe physical injury during the same time. Beyond this, it is difficult to know if there is a particular reason why participants with higher frequency of victims of severe physical injury during the last 12 months would be more inclined not to report when this was not significant for the rest of their career or for not reporting regarding perpetrators.

Overall, these findings could be interpreted to mean that, as participants have more cases, they also have more opportunities to choose not to report. Additionally, it might be hypothesized that the more cases professionals have dealt with, the more desensitized they become to the severity of violence, and hence choose not to report more so than those with less experience. However, we do not have any empirical grounds for this suggestion. Or, it could be a sign of heavy workload leading to fewer opportunities for evaluations of MR-IPV. This, in turn, could cause secondary victimization due to lack of adequate action from the institution or non-action specifically (Secondary Victimization; [11]).

Perception of compliance with MR-IPV among colleagues significantly decreased the odds ratio of choosing not to report regarding a perpetrator during the last 12 months. It is difficult to explain why this only occurred for cases during the last 12 months. However, it is intuitively understandable that if the impression of compliance among colleagues was low the participants may perceive themselves as less obligated to use MR-IPV. This also aligns with social psychological theories of group influence on individual decision making, for instance normative influence [18] which suggests individuals will conform to the group norm in order to avoid social disapproval.

Finally, as in the compliance with consent, time spent with patients, relatives, the public etc. had a decreased odds ratio of choosing not to report a perpetrator throughout career. Immediately, this might seem contradictory, however, it might still follow the same logic because it still could be that professionals find they do not have time enough with patients to evaluate the risk of IPV and consequent use of MR-IPV. The question for choosing not to report states that the participant had the knowledge of IPV risk and evaluated reporting but decided not to. If professionals do not have adequate time to evaluate risk of IPV, they will not be in a position to choose not to report.

### Strengths and limitations

The current study has several strengths and limitations. Within the large sample size, it was feasible to obtain a variety of professionals who worked with IPV victims, perpetrators, or both. Consequently, the results displayed a broad perspective on the help-services that IPV victims and perpetrators might come across and their behavior regarding MR-IPV. The study also sought to include a representative sample for both gender and geographical inclusivity. The sample covers all counties within the country, which means data from counties with mostly rural and urban communities were obtained, as well as areas with higher and lower numbers of inhabitants than the national average. This also ensured that participants with foreign origins were well represented, as they would be more concentrated around larger cities.

The research on compliance with mandatory reporting and MR-IPV is limited, which did not provide strong ground for selecting a smaller set of theory-informed independent variables or particular types of analyses. Consequently, the study was largely explorative in selection of aims, research questions, and analyses, as this was early phase research. The questionnaire, for instance, included a section on expectations of MR-IPV which was taken from a validated questionnaire and subsequently adjusted. This was done in lack of other options for validated items on this topic. However, analyses were not conducted to validate the adjusted items and this must be taken into account. Notably, it is quite possible that there are other variables that were not covered in the questionnaire or were not included in the analyses that could explain the variance in the results that were not accounted for. Additionally, this might be why some of the results are difficult to explain and interpret, and qualitative research would help to explore the processes behind such results.

In addition, the variables were measured cross-sectionally. Hence, we cannot infer a cause-and-effect relationship, as one might be able to measure with a longitudinal study. Also, the questionnaire did not measure the participants’ reasons for the decision-making about either complying or choosing not to report. Consequently, we cannot infer that they followed the law accordingly, or whether reasonable risk of violence was present or not. We would also like to acknowledge that due to the number of analyses in our study there is an increased likelihood of type I errors (e.g., [4]). Nonetheless, given the paucity of research on this topic, the present findings add valuable information to the field, for future research to build upon.

The legal context for our study was situated within the Norwegian legislation, which is, in some ways, unique compared to other legislation. For instance, “averting through other means” [39] is not typically part of mandatory reporting legislation in other countries. As such, compliance with the law among Norwegian professionals does not necessarily equal compliance with mandatory reporting among professionals under legislative frameworks in other countries. Additionally, Norway has a largely public health system, wherein both the majority of our participants were employed, and the victims and perpetrators would seek help. The increased accessibility and limited financial cost of this system to help-seekers would, arguably, influence the frequency of help-seeking among both the victims and perpetrators. Because of this, the results might not be generalizable to other countries with different health systems. Similarly, there undoubtedly may be other cultural factors that are not accounted for in our study.

In addition, we acknowledge that the dependent variables are solely relying on the participant’s self-report of compliance and choosing not to report, and we do not have data on the specific cases where they might have complied or chosen not to report. This is especially important regarding the variable for choosing not to report, because given the room of discretion within the Norwegian law (averting through other means), we cannot say with complete certainty that they did not follow the law. They certainly could have made an evaluation that was within the law, or they may not have. Because of the lack data within our study to explore this complexity, we encourage future research to explore this further.

### Implications

Compliance with MR-IPV by professionals who work with IPV victims and perpetrators is essential for the prevention of IPV. However, previous research has shown that professionals express barriers to following their mandated duty. As such, it is important to examine which characteristics either inhibit or facilitate professionals’ compliance with the law, on one hand, and choosing not to report, on the other. These findings underscore the importance of integrating instruction on MR-IPV, alongside confidentiality practices, into the curricula of relevant educational programs. Some findings in this study, however, were unexpected and difficult to explain, hence we encourage future research to explore these further to investigate the validity of these findings in other contexts. The current research may help to shape future research efforts, ultimately for the purpose of understanding compliance of MR-IPV and the prevention of IPV.

## Conclusion

The current study has contributed to the limited research on MR-IPV and compliance with MR-IPV. Our main findings suggest that there are several characteristics and factors that are associated with professionals’ compliance with the law, which is important for the sake of preventing IPV and IPH. Furthermore, our research emphasizes the importance of researching characteristics that might influence those who are mandated to report IPV to comply with their duty, or what influences them to choose not to report.

## Supplementary Information

Additional file 1. Descriptive results and translated survey: Tables for descriptive results and the translated survey used in the study.

## Data Availability

The datasets generated and/or analyzed during the current study are not publicly available due to confidentiality requirements, but generated data are available from the corresponding author on reasonable request.

## Abbreviations

MR-IPV: Mandatory reporting of intimate partner violence
IPV: Intimate partner violence
IPH: Intimate partner homicide
WHO: World Health Organization
TPB: Theory of Planned Behavior
REK: Regional Committees for Medical Research Ethics
