# Supplementary material for: Compliance with mandatory reporting of intimate partner violence among professionals in Norway
Source: BMC Public Health. 2025 May 6;25:1664. doi: 10.1186/s12889-025-22637-z (PMC12054183; doi:10.1186/s12889-025-22637-z)
Supplement: Supplementary file 1 — Additional file 1. Descriptive results and translated survey: Tables for descriptive results and the translated survey used in the study. [file 12889_2025_22637_MOESM1_ESM.docx]

**Table S-1**

*Frequency and Valid Percentages of Responses to Compliance with Mandatory Reporting of Intimate Partner Violence and Choosing Not to Report Regarding Victims and Perpetrators During the Last 12 Months and Throughout Career*

| Responses of compliance with mandatory reporting of intimate partner violence with and without consent | | | | | | | | | | | | | | |
| --- | --- | --- | --- | --- | --- | --- | --- | --- | --- | --- | --- | --- | --- | --- |
| Item | | Never *n* (%) | | Once *n* (%) | A few times (2-4 times) *n* (%) | | | Several times (5-10) *n* (%) | Many times (more than 10) *n* (%) | | | Not relevant for me *n* (%) | Missing *n* (%) | |
| Compliance w/o consent | | 156 (44.4) | | 43 (12.3) | 61 (17.4) | | | 30 (8.5) | 20 (5.7) | | | 41 (11.7) | 6 (1.7) | |
| Compliance w/ consent | | 136 (38.9) | | 32 (9.1) | 55 (15.7) | | | 39 (11.1) | 41 (11.7) | | | 47 (13.4) | 7 (2) | |
| Responses of choosing not to report reported in *n* and valid percentages | | | | | | | | | | | | | | |
| Victim | | | Yes *n* (%) | | | | No *n* (%) | | | | Missing *n* (%) | | |  |
|  | Last 12 months | | 59 (17) | | | | 288 (83) | | | | 10 (2.8) | | |  |
|  | Throughout career | | 104 (30.3) | | | | 239 (69.7) | | | | 14 (3.9) | | |  |
| Perpetrator | | |  | | |  | | | |  | | | | |
|  | Last 12 months | | 10 (2.8) | | | | 330 (92.4) | | | | 17 (4.8) | | |  |
|  | Throughout career | | 28 (8.5) | | | | 303 (91.5) | | | | 26 (7.3) | | |  |

**Table S-2**

*Frequency and Valid Percentages of Responses for Items About Perception and Knowledge of Mandatory Reporting of Intimate Partner Violence*

| Responses of participants’ expected consequences of own professional compliance with mandatory reporting of intimate partner violence | | | | |  |
| --- | --- | --- | --- | --- | --- |
| Item | No *n* (%) | Unsure *n* (%) | Yes *n* (%) | Missing *n* (%) | |
| The incident would have been reported to the supervisory health authorities | 16 (4.8) | 157 (47.1) | 160 (48) | 24 (6.7) | |
| The incident would have been reviewed at the workplace | 17 (5) | 60 (17.8) | 260 (77.2) | 20 (5.6) | |
| I would have been reproached by the patient/client/user/relatives afterwards | 69 (20.5) | 250 (74.2) | 18 (5.3) | 20 (5.6) | |
| The patient/client/user would have less trust in me | 72 (21.4) | 237 (70.3) | 28 (8.3) | 20 (5.6) | |
| The patient/client/user would have created a less trusting relationship with the support system | 59 (17.5) | 251 (74.5) | 27 (8) | 20 (5.6) | |
| There is a high probability that it would have had positive consequences for the patient/client/user | 3 (0.9) | 162 (47.9) | 173 (51.2) | 19 (5.3) | |
| There is a high probability that it would have had negative consequences for the patient/client/user | 97 (28.9) | 221 (65.8) | 18 (5.4) | 21 (5.9) | |
| All in all, the patient/client/user would have been better off | 4 (1.2) | 165 (48.8) | 169 (50) | 19 (5.3) | |
| It would have had few consequences for my patient/client/user | 63 (19) | 194 (58.4) | 75 (22.6) | 25 (7) | |
| I am very unsure what consequences it would have had for my patient/client/user | 63 (19) | 143 (43.1) | 126 (38) | 25 (7) | |
| The MR-IPV case would have made it more difficult to work afterwards | 225 (67.2) | 103 (30.7) | 7 (2.1) | 22 (6.2) | |
| The MR-IPV case would have had a negative impact on my private life | 245 (73.1) | 87 (26) | 3 (0.9) | 22 (6.2) | |
| The MR-IPV case would have made me a more secure professional | 16 (4.8) | 141 (42.3) | 176 (52.9) | 24 (6.7) | |
| The MR-IPV case would have made me a more fearful professional | 236 (70.7) | 89 (26.6) | 9 (2.7) | 23 (4.7) | |
| The MR-IPV case would have few consequences for me personally | 139 (41.6) | 120 (35.9) | 75 (22.5) | 23 (6.4) | |
| I would have received good and adequate support from the leaders at my workplace | 10 (3) | 64 (19) | 262 (78) | 21 (5.9) | |
| I would have received good and adequate support from colleagues | 8 (2.4) | 41 (12.2) | 286 (85.4) | 22 (6.2) | |
| The recipient of the message would have followed up on the message thoroughly | 5 (1.5) | 205 (61.2) | 125 (37.3) | 22 (6.2) | |
| I would have been confident that what I did was right | 8 (2.4) | 104 (31.1) | 222 (66.5) | 23 (6.4) | |

**Table S-2**

*(Continued)*

| Participants’ self-reported knowledge of mandatory reporting of intimate partner violence | | | | | | |  |
| --- | --- | --- | --- | --- | --- | --- | --- |
| Item | | No *n* (%) | To some degree *n* (%) | | Yes *n* (%) | Missing *n* (%) |  |
| Do you know the law of MR? | | 35 (9.9) | 211 (59.9) | | 106 (30.1) | 5 (1.4) |  |
| Do you know the law of MR in your field? | | 25 (7.1) | 148 (42.2) | | 178 (50.7) | 6 (1.7) |  |
| Are you informed about the criteria that should be used as the basis for evaluating the application of MR within your field? | | 37 (11) | 170 (50.4) | | 130 (38.6) | 20 (5.6) |  |
| Participants’ perceived applicability of mandatory reporting in cases of intimate partner violence in their professional practice | | | | | | |  |
| Number of cases | Victim *n* (%) | | | Perpetrator *n* (%) | | | |
| 0 | 104 (29.9) | | | 184 (54) | | | |
| 1-5 | 131 (37.6) | | | 101 (29.6) | | | |
| 6-10 | 31 (8.9) | | | 14 (4.1) | | | |
| 11-20 | 27 (7.8) | | | 16 (4.7) | | | |
| 21-30 | 16 (4.6) | | | 7 (2.1) | | | |
| 31-40 | 13 (3.7) | | | 9 (2.6) | | | |
| 41-50 | 7 (2) | | | 3 (0.9) | | | |
| 51-60 | 4 (1.1) | | | 2 (0.6) | | | |
| 61-70 | 5 (1.4) | | | 2 (0.6) | | | |
| 71-80 | 1 (0.3) | | | 0 | | | |
| 81-90 | 2 (0.6) | | | 0 | | | |
| 91-100 | 1 (0.3) | | | 1 (0.3) | | | |
| 100+ | 6 (1.7) | | | 2 (0.6) | | | |
| Missing | 9 (2.5) | | | 16 (4.5) | | | |

**Table S-3**

*Frequency and Valid Percentages of Responses for Items About Participants’ Own Workplace Conditions*

| Participants’ perceptions of mandatory reporting of intimate partner violence compliance by others | | | | | | | | | | | | |
| --- | --- | --- | --- | --- | --- | --- | --- | --- | --- | --- | --- | --- |
| Item | | No *n* (%) | | | To some degree *n* (%) | | Yes *n* (%) | | | | Missing *n* (%) | |
| In general | | 6 (1.8) | | | 65 (19.3) | | 266 (78.9) | | | | 20 (5.6) | |
| Your leaders | | 6 (1.8) | | | 47 (14) | | 282 (84.2) | | | | 22 (6.2) | |
| Your colleagues | | 9 (2.7) | | | 53 (15.8) | | 273 (81.5) | | | | 22 (6.2) | |
| Other agencies | | 15 (4.5) | | | 180 (54.4) | | 136 (41.1) | | | | 26 (7.3) | |
| Participants’ perception of workplace support | | | | | | | | | | | | |
| Item | | | Applies well *n* (%) | Applies moderately *n* (%) | | Applies poorly  n (%) | | Does not apply *n* (%) | Not applicable *n* (%) | | | Missing  *n* (%) |
| It is easy to bring up professional issues for discussion | | | 233 (74) | 69 (21.9) | | 10 (3.2) | | 2 (0.6) | 1 (0.3) | | | 42 (11.8) |
| It is okay to talk about/discuss/deliberate professional disagreements | | | 207 (65.7) | 90 (28.6) | | 16 (5.1) | | 1 (0.3) | 1 (0.3) | | | 42 (11.8) |
| They deal with disagreements appropriately | | | 160 (51.1) | 133 (42.5) | | 18 (5.8) | | 1 (0.3) | 1 (0.3) | | | 44 (12.3) |
| It is difficult to bring up unacceptable ethical behavior of colleagues | | | 41 (13.1) | 137 (43.9) | | 62 (19.9) | | 57 (18.3) | 15 (4.8) | | | 45 (12.6) |
| It is difficult to bring up unacceptable professional behavior of colleagues | | | 41 (13.1) | 137 (43.8) | | 68 (21.7) | | 55 (17.6) | 12 (3.8) | | | 44 (12.3) |
| Participants’ perceptions of their own workplace time management* | | | | | | | | | | | | |
| Item |  | | | | | | | | | *M* (*SD*) | | Missing *n* (%) |
| Work in contact with patients/users/the public (all direct contact with patients/users/clients/members of the public or their relatives, including phone calls etc.) | | | | | | | | | | 5.3 (1.7) | | 46 (12.9) |
| Meeting activities (interdisciplinary team meetings, journal meetings, supervision meetings, meetings with other agencies, etc.) | | | | | | | | | | 3.6 (1.6) | | 47 (13.1) |
| Paperwork, phone calls, emails (patient records, reports, certificates, discharge summaries, etc.) | | | | | | | | | | 4.2 (1.6) | | 48 (13.4) |
| Professional development | | | | | | | | | | 3 (1.3) | | 52 (14.6) |
| Inappropriate organizational or practical conditions, such as calling other agencies without achieving contact/arrangements, inefficient logistics, poor or lacking IT solutions, inadequate card solutions, etc. | | | | | | | | | | 2.5 (1.4) | | 52 (14.6) |
| Organizational tasks you consider unnecessary, such as filling out documents again, searching for relevant documents (records, reports, notes, etc.), reporting, unnecessary meetings, etc. | | | | | | | | | | 2.5 (1.4) | | 52 (14.6) |

*Note. *Scale of 1 to 7, “No time” = 1, “A lot of time” = 7.*

**Table S-4**

*Frequency and Valid Percentages of Responses for Items About Participants’ Experience with Intimate Partner Violence and Risk Assessment*

| Participants’ experience with cases of victims and perpetrators of intimate partner violence by type of intimate partner violence and time of case*† | | | | | | | | | |
| --- | --- | --- | --- | --- | --- | --- | --- | --- | --- |
|  | | Victim | | | | Perpetrator | | | |
| Number of cases | | Throughout career *n* (%) | | Last 12 months *n* (%) | | Throughout career *n* (%) | | Last 12 months *n* (%) | |
| Intimate partner violence | |  | |  | |  | |  | |
|  | 0 | 1 (0.3) | | 44 (12.5) | | 32 (9.1) | | 105 (30.2) | |
|  | 1-20 | 110 (31.3) | | 203 (57.5) | | 172 (49) | | 210 (60.3) | |
|  | 21-50 | 69 (19.6) | | 65 (18.4) | | 64 (12) | | 27 (7.6) | |
|  | 51-80 | 27 (7.7) | | 13 (3.7) | | 28 (7.9) | | 3 (0.9) | |
|  | 81-100+ | 145 (41.2) | | 28 (7.9) | | 55 (15.7) | | 3 (0.9) | |
| Severe intimate partner violence | |  | |  | |  | |  | |
|  | 0 | 14 (4) | | 76 (21.7) | | 65 (18.6) | | 176 (50.1) | |
|  | 1-20 | 190 (54.4) | | 245 (70) | | 207 (59.1) | | 168 (47.9) | |
|  | 21-50 | 76 (21.7) | | 17 (4.9) | | 47 (13.4) | | 4 (1.1) | |
|  | 51-80 | 25 (7.2) | | 7 (2) | | 13 (3.7) | | 2 (0.6) | |
|  | 81-100+ | 44 (12.6) | | 5(1.4) | | 18 (5.1) | | 2 (0.6) | |
| Severe physical injury | |  | |  | |  | |  | |
|  | 0 | 42 (12) | | 134 (38.4) | | 98 (27.9) | | 216 (61.2) | |
|  | 1-20 | 231 (65.8) | | 200 (57.3) | | 209 (59.5) | | 131 (37.1) | |
|  | 21-50 | 41 (11.7) | | 11 (3.2) | | 24 (6.8) | | 4 (1.1) | |
|  | 51-80 | 15 (4.3) | | 2 (0.6) | | 6 (1.7) | | 1 (0.3) | |
|  | 81-100+ | 22 (6.3) | | 2 (0.6) | | 14 (3.9) | | 1 (0.3) | |
| Participants’ experience with use of risk assessment for intimate partner violence | | | | | | | | | |
| Item | | No *n* (%) | Once *n* (%) | | More than once *n* (%) | | Unsure *n* (%) | | Missing *n* (%) |
| Some form of risk assessment | | 39 (12) | 27 (8.3) | | 240 (73.6) | | 20 (6.1) | | 31 (8.7) |
| Structured risk assessment | | 137 (42) | 23 (7.1) | | 153 (46.9) | | 13 (4) | | 31 (8.7) |

*Note. **Missing *n* (valid %): Intimate partner violence, victim, throughout career: 5 (1.4); Intimate partner violence, victim, last 12 months: 4 (1.1); Intimate partner violence, perpetrator, throughout career: 6 (1.7); Intimate partner violence, perpetrators, last 12 months: 9 (2.5); Severe intimate partner violence, victim, throughout career: 8 (2.2); Severe intimate partner violence, victim, last 12 months: 7 (2); Severe intimate partner violence, perpetrator, throughout career: 7 (2); Severe intimate partner violence, perpetrator, last 12 months: 6 (1.7); Severe physical injury, victim, throughout career: 6 (1.7); Severe physical injury, victim, last 12 months: 8 (2.2); Severe physical injury, perpetrator, throughout career: 6 (1.7); Severe physical injury, perpetrator, last 12 months: 4 (1.1). †Variable was measured in intervals of five from 1 to 10, then ten cases up to 100+ (i.e., 0, 1-5, 6-10, 11-20, 21.30, etc.), for simplicity in presentation the numbers have been collapsed in this table.

**Translated survey**

|  | Never | Once | A few times | Several times | Many times | Not relevant for me |
| --- | --- | --- | --- | --- | --- | --- |
| Have you ever complied with mandatory reporting against your patient’s/client’s/user’s wishes/without their consent? |  |  |  |  |  |  |
| Have you ever complied with mandatory reporting with the consent of the patient/client/user? |  |  |  |  |  |  |

|  | No | Yes |
| --- | --- | --- |
| Over the past 12 months, have you as a professional complied with a patient's/client's/user's wish not to report or otherwise intervene to prevent intimate partner violence, even though you were unsure whether the patient/client/user understood the risk of violence related to their own situation? | | |
| Working with a victim |  |  |
| Working with a perpetrator |  |  |
| During your career, have you as a professional complied with a patient's/client's/user's wish not to report or otherwise intervene to prevent intimate partner violence, even though you were unsure whether the patient/client/user understood the risk of violence related to their own situation? | | |
| Working with a victim |  |  |
| Working with a perpetrator |  |  |

|  | No | Unsure | Yes |
| --- | --- | --- | --- |
| The incident would have been reported to the supervisory health authorities |  |  |  |
| The incident would have been reviewed at the workplace |  |  |  |
| I would have been reproached by the patient/client/user/relatives afterwards |  |  |  |
| The patient/client/user would have less trust in me |  |  |  |
| The patient/client/user would have created a less trusting relationship with the support system |  |  |  |
| There is a high probability that it would have had positive consequences for the patient/client/user |  |  |  |
| There is a high probability that it would have had negative consequences for the patient/client/user |  |  |  |
| All in all, the patient/client/user would have been better off |  |  |  |
| It would have had few consequences for my patient/client/user |  |  |  |
| I am very unsure what consequences it would have had for my patient/client/user |  |  |  |
| The MR-IPV case would have made it more difficult to work afterwards |  |  |  |
| The MR-IPV case would have had a negative impact on my private life |  |  |  |
| The MR-IPV case would have made me a more secure professional |  |  |  |
| The MR-IPV case would have made me a more fearful professional |  |  |  |
| The MR-IPV case would have few consequences for me personally |  |  |  |
| I would have received good and adequate support from the leaders at my workplace |  |  |  |
| I would have received good and adequate support from colleagues |  |  |  |
| The recipient of the message would have followed up on the message thoroughly |  |  |  |
| I would have been confident that what I did was right |  |  |  |

|  | No | To some extent | Yes |
| --- | --- | --- | --- |
| Do you know the law of MR? |  |  |  |
| Do you know the law of MR in your field? |  |  |  |
| Are you informed about the criteria that should be used as the basis for evaluating the application of MR within your field? |  |  |  |

|  | 0 | 1-5 | 6-10 | 11-20 | 21-30 | 31-40 | 41-50 | 51-60 | 61-70 | 71-80 | 81-90 | 91-100 | 100+ |
| --- | --- | --- | --- | --- | --- | --- | --- | --- | --- | --- | --- | --- | --- |
| In how many of the cases of intimate partner violence where you encountered individuals exposed to intimate partner violence do you feel that mandatory reporting was triggered? |  |  |  |  |  |  |  |  |  |  |  |  |  |
| In how many of the cases of intimate partner violence where you encountered individuals who perpetrated intimate partner violence against others do you feel that mandatory reporting was triggered? |  |  |  |  |  |  |  |  |  |  |  |  |  |

|  | 0 | 1-5 | 6-10 | 11-20 | 21-30 | 31-40 | 41-50 | 51-60 | 61-70 | 71-80 | 81-90 | 91-100 | 100+ |
| --- | --- | --- | --- | --- | --- | --- | --- | --- | --- | --- | --- | --- | --- |
| In your professional practice, how many people have you encountered who have been victims of intimate partner violence? | | | | | | | | | | | | | |
| During your career |  |  |  |  |  |  |  |  |  |  |  |  |  |
| The last 12 months |  |  |  |  |  |  |  |  |  |  |  |  |  |
| In your professional practice, how many people have you encountered who have perpetrated intimate partner violence? | | | | | | | | | | | | | |
| During your career |  |  |  |  |  |  |  |  |  |  |  |  |  |
| The last 12 months |  |  |  |  |  |  |  |  |  |  |  |  |  |
| In your professional practice, how many people have you encountered who have been subjected to severe intimate partner violence (high potential for harm and/or persistent violence)? | | | | | | | | | | | | | |
| During your career |  |  |  |  |  |  |  |  |  |  |  |  |  |
| The last 12 months |  |  |  |  |  |  |  |  |  |  |  |  |  |
| In your professional practice, how many people have you encountered who have perpetrated severe intimate partner violence (high potential for harm and/or persistent violence)? | | | | | | | | | | | | | |
| During your career |  |  |  |  |  |  |  |  |  |  |  |  |  |
| The last 12 months |  |  |  |  |  |  |  |  |  |  |  |  |  |
| In your professional practice, how many people have you encountered who have been severely physically injured as a result of intimate partner violence? | | | | | | | | | | | | | |
| During your career |  |  |  |  |  |  |  |  |  |  |  |  |  |
| The last 12 months |  |  |  |  |  |  |  |  |  |  |  |  |  |
| In your professional practice, how many people have you encountered who have caused severely physically injured as a result of intimate partner violence? | | | | | | | | | | | | | |
| During your career |  |  |  |  |  |  |  |  |  |  |  |  |  |
| The last 12 months |  |  |  |  |  |  |  |  |  |  |  |  |  |

|  | No | To some extent | Yes |
| --- | --- | --- | --- |
| If life and health is at stake, is it your impression that mandatory reporting is usually complied with? |  |  |  |
| If life and health is at stake, is it your impression that mandatory reporting is usually complied with by your leaders? |  |  |  |
| If life and health is at stake, is it your impression that mandatory reporting is usually complied with by your colleagues? |  |  |  |
| If life and health is at stake, is it your impression that mandatory reporting is usually complied with by other help services? |  |  |  |

|  | Applies well | Applies moderately | Applies poorly | Does not apply | Not applicable |
| --- | --- | --- | --- | --- | --- |
| It is easy to bring up professional issues for discussion |  |  |  |  |  |
| It is okay to talk about/deliberate/discuss professional disagreements |  |  |  |  |  |
| We handle professional disagreements in an appropriate manner. |  |  |  |  |  |
| It is difficult to address unacceptable ethical behavior of colleagues |  |  |  |  |  |
| It is difficult to address unacceptable professional work of colleagues |  |  |  |  |  |

|  | No time (1) | | | | A lot of time (7) | | | |
| --- | --- | --- | --- | --- | --- | --- | --- | --- |
| Work in contact with patients/users/the public (all direct contact with patients/users/clients/members of the public or their relatives, including phone calls etc.) |  |  |  |  | |  |  |  |
| Meeting activities (interdisciplinary team meetings, journal meetings, supervision meetings, meetings with other agencies, etc.) |  |  |  |  | |  |  |  |
| Paperwork, phone calls, emails (patient records, reports, certificates, discharge summaries, etc.) |  |  |  |  | |  |  |  |
| Professional development |  |  |  |  | |  |  |  |
| Inappropriate organizational or practical conditions, such as calling other agencies without achieving contact/arrangements, inefficient logistics, poor or lacking IT solutions, inadequate card solutions, etc. |  |  |  |  | |  |  |  |
| Organizational tasks you consider unnecessary, such as filling out documents again, searching for relevant documents (records, reports, notes, etc.), reporting, unnecessary meetings, etc. |  |  |  |  | |  |  |  |

|  | No | Once | More than once | Unsure |
| --- | --- | --- | --- | --- |
| Have you ever conducted any form of risk assessment for intimate partner violence? |  |  |  |  |
| Have you conducted a structured risk assessment for intimate partner violence (used a tool/form/manual)? |  |  |  |  |

| Check off for the context you were recruited from | |
| --- | --- |
| Police |  |
| Child Welfare Services |  |
| Emergency Department |  |
| Sexual Assault Centers |  |
| Anger Management |  |
| Crisis Shelters |  |
| Alternative to Violence |  |
| Retired |  |
| Other, specify: |  |

**Write how many years you have worked in your current position:** |__|__|

**Age:** |__|__|

| Gender | Man | Woman | Other, specify |
| --- | --- | --- | --- |
|  |  |  |  |
